# Performance evaluation and prediction of optimal operational conditions for a compact date seeds milling unit using feedforward neural networks

**DOI:** 10.1038/s41598-025-87508-4

**Published:** 2025-02-08

**Authors:** Khaled Abdeen Mousa Ali, Changyou Li, Wang Han, Sali Issa, Mohamed Hamdy Eid, Samy F. Mahmoud, Marwa Abd-Elnaby Mohammed

**Affiliations:** 1 College of Engineering, South China Agricultural University, Guangzhou, 510642 China; 2 College of Agricultural Engineering, Al-Azhar University, Cairo, 11651 Egypt; 3 School of Intelligent Engineering, Shaoguan University, Shaoguan, China; 4 Electrical Information of Science and Technology, Hubei University of Education, Wuhan, China; 5 Institute of Environmental Management, Faculty of Earth Science, University of Miskolc, Miskolc- Egyetemváros, 3515 Hungary; 6 Geology Department, Faculty of Science, Beni-Suef University, Beni-Suef, 65211 Egypt; 7 Department of Biotechnology, College of Science, Taif University, Taif City, Saudi Arabia; 8 Agricultural Engineering Research Institute (AENRI), Dokki, Giza Egypt

**Keywords:** Date seed processing, Feedforward neural network, Agricultural waste valorization, Milling machine design, Sustainable resource utilization, Energy infrastructure, Power distribution, Energy science and technology

## Abstract

Date seed grinding remains a significant challenge limiting the utilization of this valuable agricultural by-product." In this study, a compact date seeds grinding unit was designed, tested, and evaluated. The machine has two primary: a pair of toothed cylinders and a hammer mill. The machine’s performance was assessed in terms of throughput, specific energy consumption, and mean particle size of the product. First, the cylindrical section was tested under various conditions, including cylinder rotational speed (150, 250, 350, and 450 rpm), feed gate opening size (30, 37.5, and 45 cm^2^), and the clearance between cylinders (0, 1, and 2 mm). The feedforward neural network (FNN) framework predicated the optimal operating conditions for this part, which were recorded as 150 rpm cylinder rotational speed, 45 cm^2^ feed gate opening, and 2 mm cylinder clearance. This optimal operational condition was utilized as the starting conditions for subsequent testing of the hammer mill section. Then, the hammer mill was tested with different hammer rotational speeds (1250, 1500, and 1750 rpm) and screen hole diameters (2, 4, and 6 mm) underneath the hammers. The FNN model was again employed to predicate the most suitable operating parameters for the grinding unit. The key results included the optimal operational parameters at 150 rpm cylinder rotational speed, 2 mm clearance, 45 cm^2^ feeding area, 1750 rpm hammer speed, and 6 mm screen hole diameter. That operational condition resulted in 30 kg/h for machine’s throughput, 49 kW h/ton specific energy consumption, and 2.14 mm mean product size. With FNN model accuracy R^2^ of 0.99974, demonstrating high prediction reliability. Meanwhile, the operating cost was 0.027 $/kg, suitable for small to medium-scale operations. The significance of these findings lies in the development of an efficient, versatile milling solution for date seeds and similar agricultural materials. This research pioneers the application of machine learning in optimizing date seed processing, potentially revolutionizing agricultural waste valorization and opening new avenues for sustainable resource utilization.

## Introduction

Dates represent a high-value agricultural commodity worldwide^1^; date production, industrialization, and utilization have recently been continuously increasing, as per the Food and Agricultural Organization (FAO)^2^, which led to a rapid increase in date fruit waste and agro-waste during fruit maturation and harvesting. Storage and conditioning may also increase fruit waste^3^. Moreover, date waste causes serious environmental problems, especially greenhouse gas emissions, which contribute to global warming^3^.

Date seeds, on average, constitute approximately 5.6–14.2% of the overall fruit weight. These seeds are known for being abundant in various beneficial phytochemicals, including phenols, sterols, carotenoids, anthocyanins, procyanidins, and flavonoids. Consequently, date seeds are an excellent natural source of these health-promoting compounds^4,5^. The moisture content of date seeds varies between 8.64 and 12.25%, the proteins ranging from 1.22 to 3.30%, fats contain ranging from 4.81 to 8.77%, ash content between 0.82 and 1.14%^6,7^, carbohydrates from 2.43 to 4.65%^8^, and dietary fiber levels between 67.56 and 74.20%^9,10^. Regarding the mineral content, date seeds contain approximately 51.7 mg to 58.4 mg of magnesium per 100 g, 28.9 mg to 38.8 mg of calcium, 83.6 mg to 68.3 mg of phosphorus, 229 mg to 293 mg of potassium, 10.25 mg to 10.4 mg of sodium, and 2.30 mg to 2.21 mg of iron^9,11^. Recent studies indicated that palm-dates fruit and seed possess remarkable anti-aging properties and can effectively combat skin wrinkling in women. Additionally, it suggested that date waste contains numerous vital components that can enhance hair and skin health. These components are believed to prevent premature greying of hair, inhibit the formation of wrinkles, and contribute to a rejuvenated and revitalized appearance of the skin^10^.

Date seeds have the potential to be daily consumed as they contain approximately 4–14% oil. This oil consists of both saturated such as lauric, myristic, and palmitic acids and unsaturated fatty acids including palmitoleic, oleic, linoleic, and linolenic acids^12^. Among these, oleic acid also known as omega-9 monounsaturated fatty acid, represents C18:1, the most abundant fatty acid in date seeds and extracted oils^13^. Compared to bran oil, date seed oil is an excellent oleic acid source^14^. The oil derived from date seeds is in a liquid state at room temperature and possesses a vibrant yellow color and a pleasant aroma. It is edible and has recently gained commercial significance due to its diverse biological components and physicochemical properties. Furthermore, the oil extracted from date seeds is widely used in cosmetics. However, date seeds also contain medicinal compounds, including corticosteroids, which can potentially treat kidney bladder disorders, inflammation, and infectious diseases. Numerous studies have demonstrated the anti-inflammatory properties of date seeds^15–17^.

For efficient date seed processing, subjecting them to a grinding process is necessary to convert them into a commonly employed form. The date-seeds milling presents challenges due to its elevated cellulose content, hardness, and lack of specialized milling equipment for this valuable by-product. Ref.^18^ fabricated and evaluated the performance of a hammer and identified its optimal operational conditions. The evaluation involved testing various screen hole diameters 1.5, 2.0, and 3.0 mm, hammer tip speeds 68.12, 81.81, and 102.17 m/s, and hammer thicknesses 4.0, 5.0, and 6.0 mm to assess energy consumption and geometric mean diameter of the flour using a modified central composite design. The results showed that screen hole diameter and hammer thickness significantly influenced energy consumption. Additionally, screen hole diameter and hammer tip speed affected the geometric mean diameter, while hammer thickness did not. Ref.^19^ concluded that the grinding efficiency of hammer mills is influenced by both the properties of the material being ground and the characteristics of the hammers. Fundamental material properties affecting energy consumption include fineness grade, shape, moisture content, hardness, density, strength, porosity, abrasiveness, and stickiness.

Additionally, several factors related to the hammer mill itself play a significant role, including hammer circumferential velocity, hammer shape, feed rate, type of crusher unit, size and shape of the unit, hammer-sieve range, grinding product discharge method, the ratio of hole area to total screen area, sieve surface, geometry, and overall construction of the crusher unit. Ref.^20^ evaluated and compared the grinding operations of corn cobs using two manufactured hammer mills “Aamagro and El-Gohary”, all experiments aimed to assess the performance of both mills under varying conditions, including three feeding rates 0.2, 0.4, and 0.6 tons per hour, four different rotational speeds 1200, 1500, 1800, and 2100 rpm, and two screen hole diameters 9 and 14 mm. Performance metrics included particle size distribution, machine productivity, power requirements, specific energy consumption, and machinery unit cost. The results indicated that the optimal operating conditions for both mills were at a rotor speed of 2100 rpm, a feeding rate of 0.2 tons per hour, and a screen hole diameter of 9 mm.

Deep learning encompasses computationally intensive models like fully connected multi-layer neural networks, requiring precise evaluation of numerous parameters and substantial training data^21^. While more complex architectures like Convolutional Neural Networks (CNN) and Deep Neural Networks (DNN) have demonstrated superior performance in various applications, FNN’s simpler architecture provides efficient parameter optimization with lower computational requirements. For agricultural machinery applications where input–output relationships are primarily numerical, FNN offers an effective balance between model complexity and prediction accuracy^22^.

Deep learning encompasses computationally intensive models, such as fully connected multi-layer neural networks, which require precise evaluation of numerous parameters. A substantial amount of data is necessary for effective training. Although there are no strict guidelines for the minimum amount of training data, a commonly accepted rule is to have at least ten times as many samples as parameters in the network^23^. Ref.^24^ provided a comprehensive overview of deep learning and emphasized that FNNs are among the simpler machine learning techniques. However, they discussed the evolution of more complex architectures like CNNs and DNNs, which have significantly outperformed traditional methods in various applications. Ref.^25^ they proposed an intelligent deep neural network system for grinding wheel condition monitoring. While Ref.^26^ used neural networks, support vector machines, and K-nearest neighbor machine learning methods for wheel monitoring. In Ref.^27^, they depended on a radial basis neural network for gear grinding machine performance analysis. As well, Ref.^28^ utilized a support vector machine for a grinder prediction analysis. Recent advances in transfer learning techniques have significantly improved grinding tool condition monitoring, achieving over 90% tool lifetime utilization through accurate prediction of remaining useful life. This approach demonstrates superior performance in addressing data distribution and deficiency issues, with encoding metric-based model selection showing a strong correlation “Pearson coefficient of 0.79” for optimizing grinding processes^29^. Recent research has demonstrated the effectiveness of hybrid machine learning approaches in grinding tool condition monitoring, combining traditional statistical features with physical modeling to achieve R^2^ scores exceeding 0.95. By integrating sensor data with kinematic geometrical process models, these systems can accurately predict the remaining tool lifetime and optimize grinding operations, significantly advancing process monitoring and control^30^. Researchers of^31^ demonstrated the effectiveness of machine learning in predicting grinding outcomes for complex materials, with Gradient Boosting and EfficientNet models achieving high accuracy in predicting surface roughness and grinding forces. Integrating ML techniques with grinding force prediction provides a comprehensive approach to process monitoring and optimization, highlighting the growing potential of AI-driven solutions in grinding applications.

FNN is a computational architecture for processing complex data using multiple interconnected processors and computational paths. Artificial neural networks, created by analogy with the human brain, can train and analyze large, complex data sets that are extremely difficult to process using more linear algorithms. Furthermore, it can make accurate decisions and predictions more than the traditional statistical and mathematical models^32^. Ref.^33^ in their study which included a total of 192 samples, of which 128 were allocated for calibration and 64 for prediction. The FNN model effectively estimated nitrogen levels, attaining a coefficient of determination R^2^ of 0.903 for the prediction set. The performance of the FNN has been enhanced by integrating it with the Emperor Penguin Optimization (EPO) algorithm, which refines the model’s parameters for improved accuracy in predictions based on a dataset of energy meter readings from 1,636 unique buildings^34^. Ghosh and Maji^35^ evaluate various machine learning algorithms for applications in precision agriculture, emphasizing their effectiveness in predicting agricultural outcomes. Among the algorithms assessed, FNN is highlighted as one of the traditional machine learning techniques. While FNNs are useful for predictive modeling, the authors pointed out that they are relatively simpler compared to more advanced techniques such as DNN and CNN. They illustrated that FNNs, despite their capabilities, lack the complexity and depth of these newer models, which can capture more intricate patterns in data due to their multi-layered architectures and advanced learning algorithms. Their comparison showed that while FNNs can provide satisfactory results in certain scenarios, they do not leverage the same level of sophistication as deep learning models, which can lead to improved accuracy and performance in complex agricultural datasets. Their findings suggested that for more nuanced predictions, especially in high-dimensional data contexts, adopting deeper learning techniques may yield better results than relying solely on FNNs. While deep learning techniques like CNNs and DNNs can provide superior performance in complex pattern recognition tasks, the selection of FNN for this study was driven by practical considerations. The relatively simple numerical relationships between operational parameters and performance metrics in milling operations, combined with our dataset size of 216 samples, made FNN an appropriate choice. The high prediction accuracy achieved R^2^ = 0.99974 validates this decision, demonstrating that FNN’s simpler architecture was sufficient for optimizing the date seeds milling process without requiring the computational complexity of deeper architectures.

Recent advances in machine learning applications for manufacturing systems have shown promising results in process optimization. Ref.^36^ achieved high prediction accuracy using geometry-integrated neural networks for metal processing, demonstrating the potential of advanced ML architectures. In precision machinery optimization, authors of^37^ implemented multi-objective approaches for cooling systems, highlighting the importance of integrated modeling solutions. Additionally, Guo et al.^38^ developed a knowledge graph-based approach for mechanical product modeling, establishing new methodologies for process information integration. Similar to these studies, our research applies machine learning techniques to optimize agricultural processing equipment, specifically focusing on date seed milling operations.

The novelty of this research lies in developing an innovative compact date seeds milling unit that combines both crushing and milling mechanisms in a single machine, along with the pioneering application of FNN to predict optimal operational parameters. Unlike existing studies focusing on conventional milling techniques, this integrated approach achieves superior efficiency with low specific energy consumption while producing customizable particle sizes. This represents the first implementation of machine learning algorithms for optimizing date seed milling operations.

**Fig. 1:**
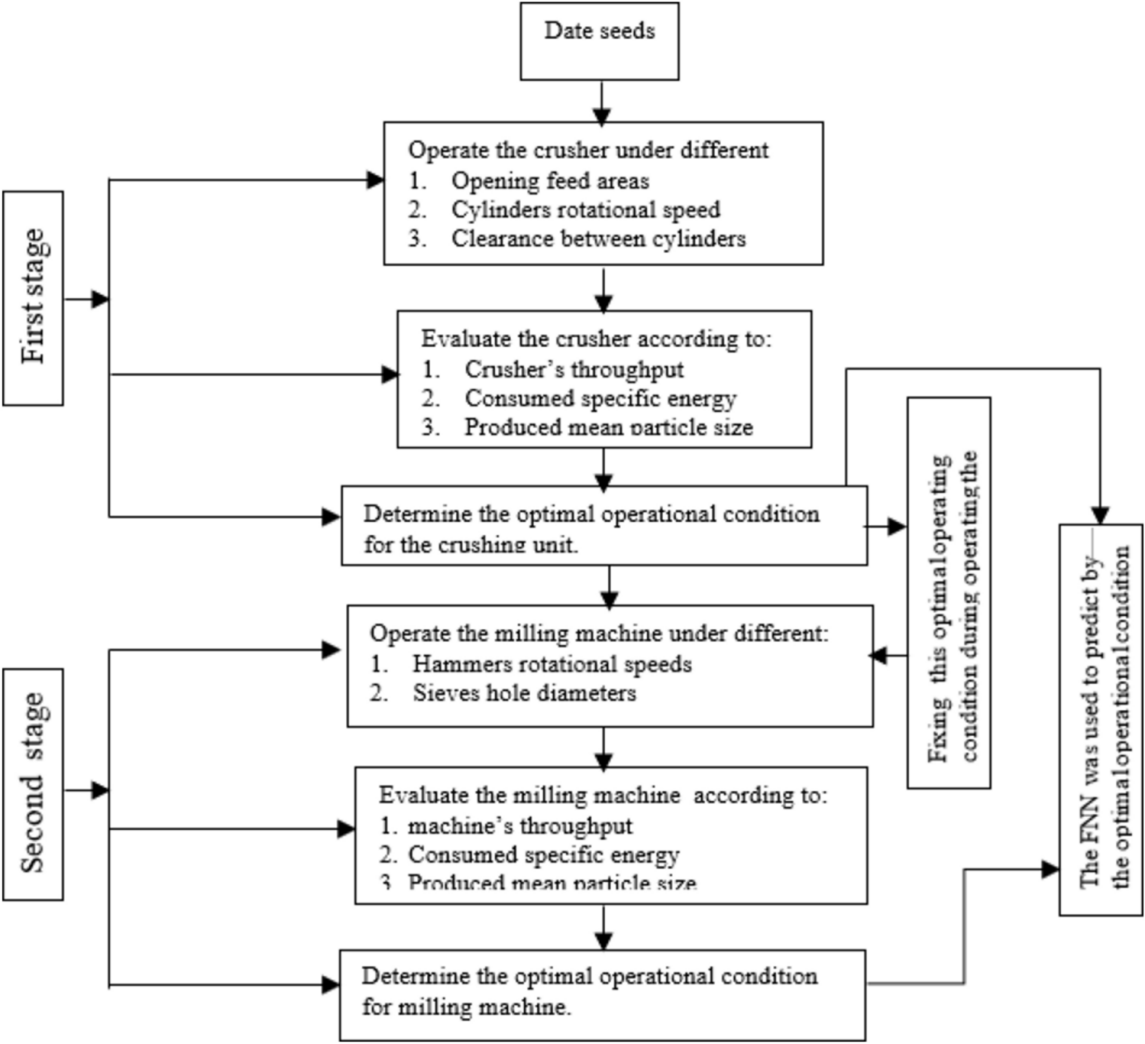
Flow chart of the research methodology for evaluating and optimizing the date seeds milling unit.

The objectives of this study were to: Design and evaluate a compact dual-stage milling machine specifically tailored for date seeds, integrating both crushing and hammer mill mechanisms. Investigate the effects of key operational parameters (cylinder speed, clearance, feeding area, hammer speed, and screen size) on machine performance metrics including throughput, specific energy consumption, and mean particle size. Develop and validate a feedforward neural network model to optimize the operational parameters for both crushing and milling stages, maximizing efficiency while maintaining product quality. This research represents the first implementation of machine learning algorithms for optimizing date seed milling operations, establishing a foundation for smart agricultural machinery development. Figure 1 presents a flow chart of the research methodology for evaluating and optimizing the date seeds milling unit.

## Materials and methods

**Fig. 2:**
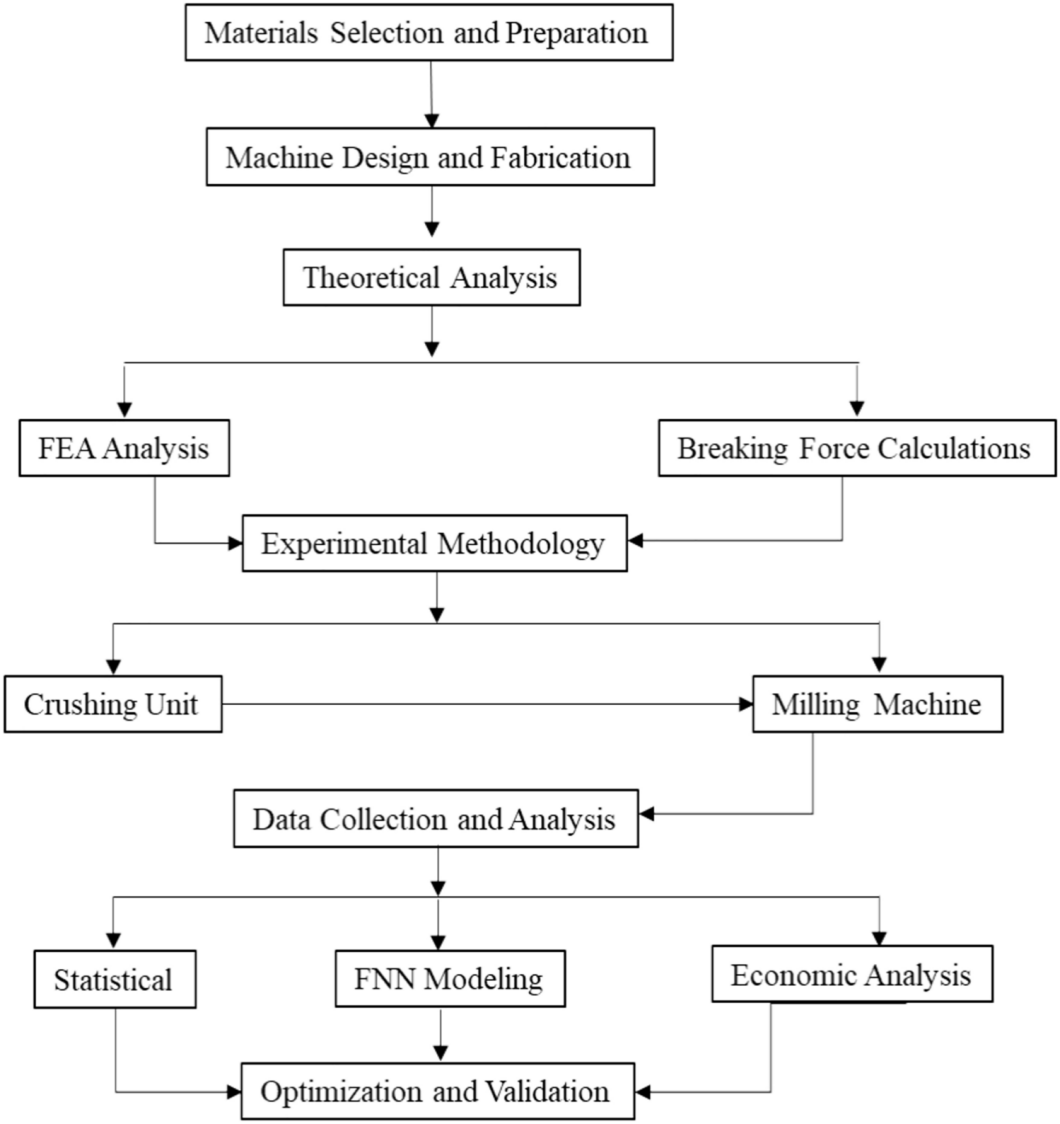
The framework of methodology.

The “Sewi” variety of date seeds was sourced from local date processing factories. Initially, the seeds were washed to remove any residual fleshy date material. After that, the seeds were naturally dried under sunlight. Subsequently, the seeds’ physical and mechanical properties were measured and considered in the machine design process. The Sewi date seeds exhibit high resistance to shear and compression forces compared to various other grains and several date seed varieties. This feature enables the designed milling machine not only to grind Sewi date seeds effectively but also to process other seeds and grains that are less resistant to shear and compression forces and have lower cellulose content^39^ The machine was fabricated and tested during the 2015–2016 period, and the findings were applied using FNN in 2024. Figure 2 illustrates this study’s framework methodology.

### Experimental design

The experimental methodology comprised a two-phase evaluation of the date seed milling apparatus. In the initial phase, the crushing unit’s performance was assessed independently to determine its optimal operational parameters. This process involved systematic testing of the crushing mechanism in isolation to identify the most efficient configuration. The subsequent phase commenced after establishing the crushing unit’s optimal settings. This second stage encompassed a comprehensive evaluation of the entire milling system, integrating both the optimized cylindrical crushing component and the hammer mill. This approach allowed for a holistic assessment of the machine’s performance under optimized conditions, ensuring the most effective synergy between the crushing and milling processes. The crushing unit was examined and evaluated under the following parameters: four different rotational speeds named 150 rpm, 250 rpm, 350 rpm, and 450 rpm equivalent to linear velocities of 6.28 m/s, 10.47 m/s, 14.65 m/s, and 18.84 m/s, respectively, three different clearances between the cylinders named 0 mm, 1 mm, and 2 mm, and three different feed gate open areas of 30 cm^2^, 37.5 cm^2^, and 45 cm^2^. Once the optimal operational condition of the crushing unit was determined, it was fixed and used as a baseline for testing the hammer mill machine “crushing unit and milling unit together”. The milling unit was examined under four different rotational speeds of hammers named 1250 rpm, 1500 rpm, and 1750 rpm equivalent to linear velocities of 23.43 m/s, 28.11 m/s, and 32.80 m/s, respectively, and three different sieves with different opening hole diameters named 2 mm, 4 mm, and 6 mm. The sieves maintained a constant perforated ratio to a sieve area of 28%. Each test was replicated three times. The experimental design aims to comprehensively evaluate the date-seeds milling machine performance by systematically varying and controlling these parameters, identifying the optimal operational conditions for crushing and hammer mill units. These evaluations are based on three key metrics: throughput kg/h, consumed specific energy kW·h/ton, and mean particle size produced mm. The controlled environment facilitated precise measurements and repeatable conditions, ensuring the results’ reliability. It is important to note that the machine’s operating principles and performance are designed to be consistent, regardless of whether it is used indoors or outdoors.

### Milling prototype

#### Power source and power transmission system

**Fig. 3:**
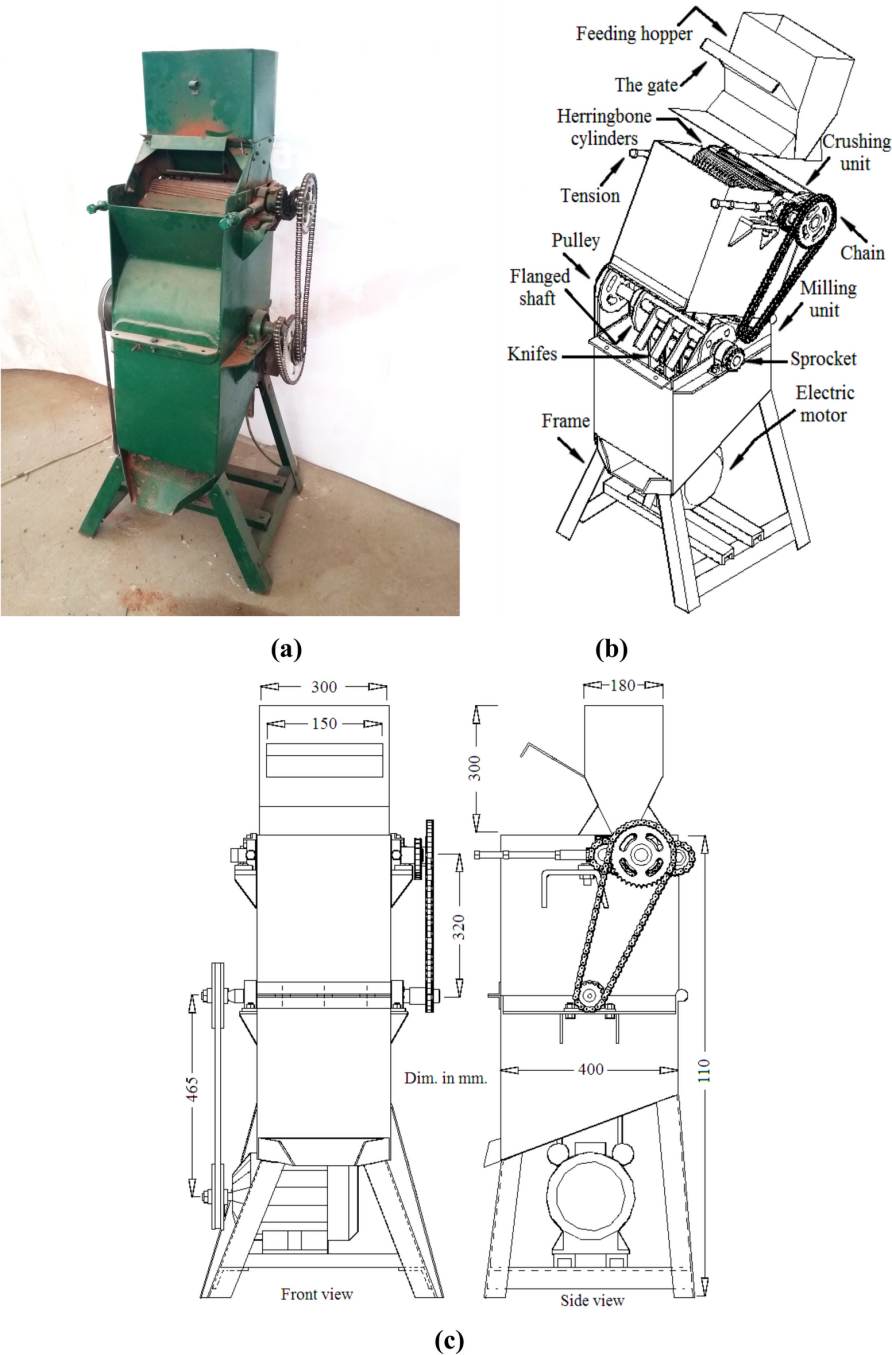
A photograph of the date seeds milling machine (**a**), a detailed drawing of its essential components (**b**), and a dimensional drawing (**c**). All dimensions are in centimeters.

Power transfer was achieved through an electric motor operating at 1400 rpm. Two pulleys and a V-belt were utilized to transmit power from the motor to the milling unit, while two sprockets and a chain were employed to convey power from the milling unit to the crushing unit, as shown in Fig. 3.

#### Feeding hopper

**Fig. 4:**
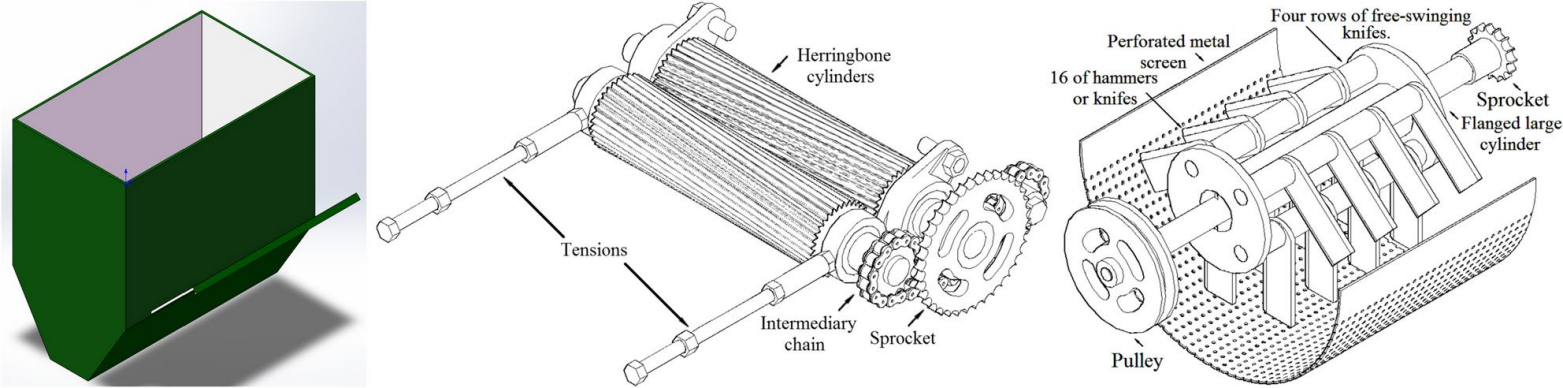
A 3D module for feeding hopper “left”, main components of the crushing part “middle”, and main components of the milling unit “right” **.**

The feeding hopper, which holds about 11 kg of date seeds, consists of two sections. The first section was constructed as a hollow box, while the second section took on a trapezoidal shape. To regulate the feeding rate of date seeds from the hopper to the crushing unit, a gate with a width of 30 cm was implemented on the side of the hopper. The friction angle between date seeds and galvanized sheets was considered during the gate design Fig. 4.

#### Crushing unit

The crushing unit served the purpose of facilitating the milling process by crushing the date seeds. This was accomplished using a pair of herringbone cylinders with dimensions of 80 mm in diameter and 280 mm in length. The cylinders were equipped with two clearance adjustment screws, enabling the modification of the gap between them Fig. 4.

#### Milling unit

The milling process was executed utilizing a hammer mill. The milling unit consists of freely movable knives mounted on four horizontal shafts. The construction material for the knives was flat spring steel, with dimensions of 114 mm in length, 45 mm in width, and 6 mm in thickness, as depicted in Fig. 4. The shafts were securely fastened to flanges on a large cylinder powered by an electric motor. The milling unit comprises a hammer mill with a substantial cylinder housing a horizontal shaft that propels a rotor with four freely oscillating knives. These hammers/knives rotate within a perforated metal screen featuring openings of 2 mm, 4 mm, and 6 mm in size.

The versatility of the machine for processing various seeds is ensured through several adjustable features. The clearance between the crushing cylinders can be modified using adjustment screws, allowing for adaptation to different seed sizes and hardness. The hammer mill’s interchangeable screens with various hole diameters 2–6 mm enable customization of the final particle size for different seed types. Additionally, the variable speed control for both the crushing cylinders and hammer mill allows for fine-tuning the milling process to accommodate seeds with different physical properties, ensuring efficient processing across a range of agricultural materials.

### Theoretical study

Due to the high energy required to mill the date seeds, the idea of this study depends on turning date seeds into broken parts using firstly a toothed pair of cylinders that rotate in opposite directions inward; theoretically, this part of the machine follows Eq. 1 to broken seeds^40^.

1 $${\text{P}} = \frac{{\text{F}}}{{\text{A}}}$$

where, P is the seed’s broken pressure N/m^2^; F is the broken deeded force N and A is the area under load m^2^. The breaking pressure calculation is essential for determining the minimum force required to initiate seed fracture, which guided the design of the toothed cylinders. This pressure value “2.551 kN/m^2^ for compression and 1.37 kN/m^2^ for shear” informed the cylinder teeth specifications and rotational speed selection to ensure efficient seed breaking while minimizing energy consumption. The broken parts of the seeds then pass to the milling chamber, where they are subjected to a milling process using 16 hammers according to the following equation^41^:

2 $${\text{I}}_{\text{impact}}=\frac{{\text{mv}}^{2}}{2}$$

where I _impact_ is the impact energy J; m is the mass of the free knife 0.25 kg; and v is the liner speed at the outer end of the knife m/s. The average shear force for crushed date seed was measured as 0.2495 kN. For Eq. 2, the impact energy was determined using the measured mass of each hammer and the calculated linear velocity at the hammer’s outer end. These calculations guided the design of the hammer mill components. The MT 2021 Universal Material Tester with 20 kN capacity and 1N accuracy was used to measure the date seed’s shear and compression forces.

**Fig. 5:**
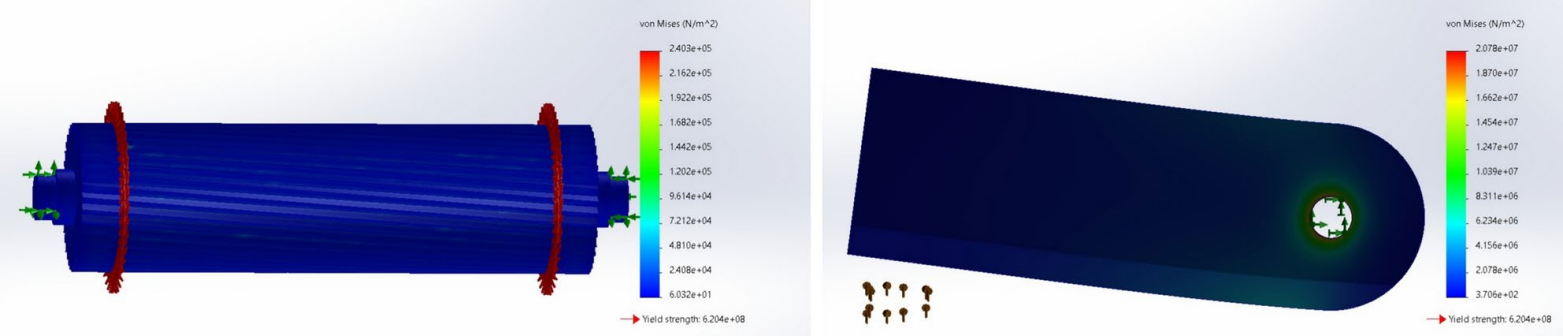
Stress Analysis of Herringbone Cylinders “left” and Hammer Knives “right” under Mechanical Stress.

The Finite Element Analysis (FEA) using SolidWorks was conducted to evaluate the mechanical stress on critical parts, including the herringbone cylinders and hammer knives as presented in Fig. 5. The simulations assessed the static stress distribution and confirmed that the components remain well within the yield strength limits under operational conditions.

The experimental methodology used a stopwatch to measure the milling time required to process 1 kg of date seeds. This measurement determined the machine’s productivity, expressed in kg/h^42^.

3 $${\text{Throughput~}}~\frac{{{\text{kg}}}}{{\text{h}}} = \frac{{{\text{mass~of~milled~or~crushed~datd~pits}}~\left( {{\text{kg}}} \right)}}{{{\text{time~of~milling~process}}~\left( {\text{h}} \right)}}$$

To calculate the consumed specific energy during experiments, the Equation number 4 was employed^43^, meanwhile, the required power was measured according to Eq. 5^44^:

4 $${\text{Specific~energy~}}\frac{{{\text{kW}}\;{\text{h}}}}{{{\text{ton}}}} = \frac{{{\text{requiered~power}}~\left( {{\text{kW}}} \right)}}{{{\text{machine~production}}~\left( {\frac{{{\text{ton}}}}{{\text{h}}}} \right)}}$$

5 $${\text{Required power}}\,\left( {{\text{kW}}} \right) = \frac{{I*V*\mu *cos\Theta }}{1000}$$

where I is the line current strength in amperes (A), V is the potential difference (V), which was equal to 220 V, η is the mechanical efficiency, which was assumed to be 90%, and cos θ is the power factor (85%).

The Sieve Shaker Minor 200 was employed to ascertain the mean particle size distribution of the output product obtained from the milling process. Accurate measurements of the total sample mass and the mass of individual product categories were obtained using a precise digital scale of 0.01 g. This meticulous weighing process ensured reliable and accurate data for analyzing the particle size distribution of the milled product. The mean particle size of the milled date seeds was determined using the equation described by^45^:

6 $$\text{d }=\frac{\sum (\text{C }\times \text{ M})}{\sum \text{ M}}$$

where d represents the mean particle size of the milled date seeds in mm; C represents the size of the milled date seeds above each sieve in mm, and M represents the mass of the milled date seeds above each sieve in g.

#### The statistical analysis

The recorded data were analyzed using SPSS software version 20 (SPSS Inc., Chicago, IL, USA), employing a two-way ANOVA test with a significance level of 5% (LSD).

#### FNN implementation

**Fig. 6:**
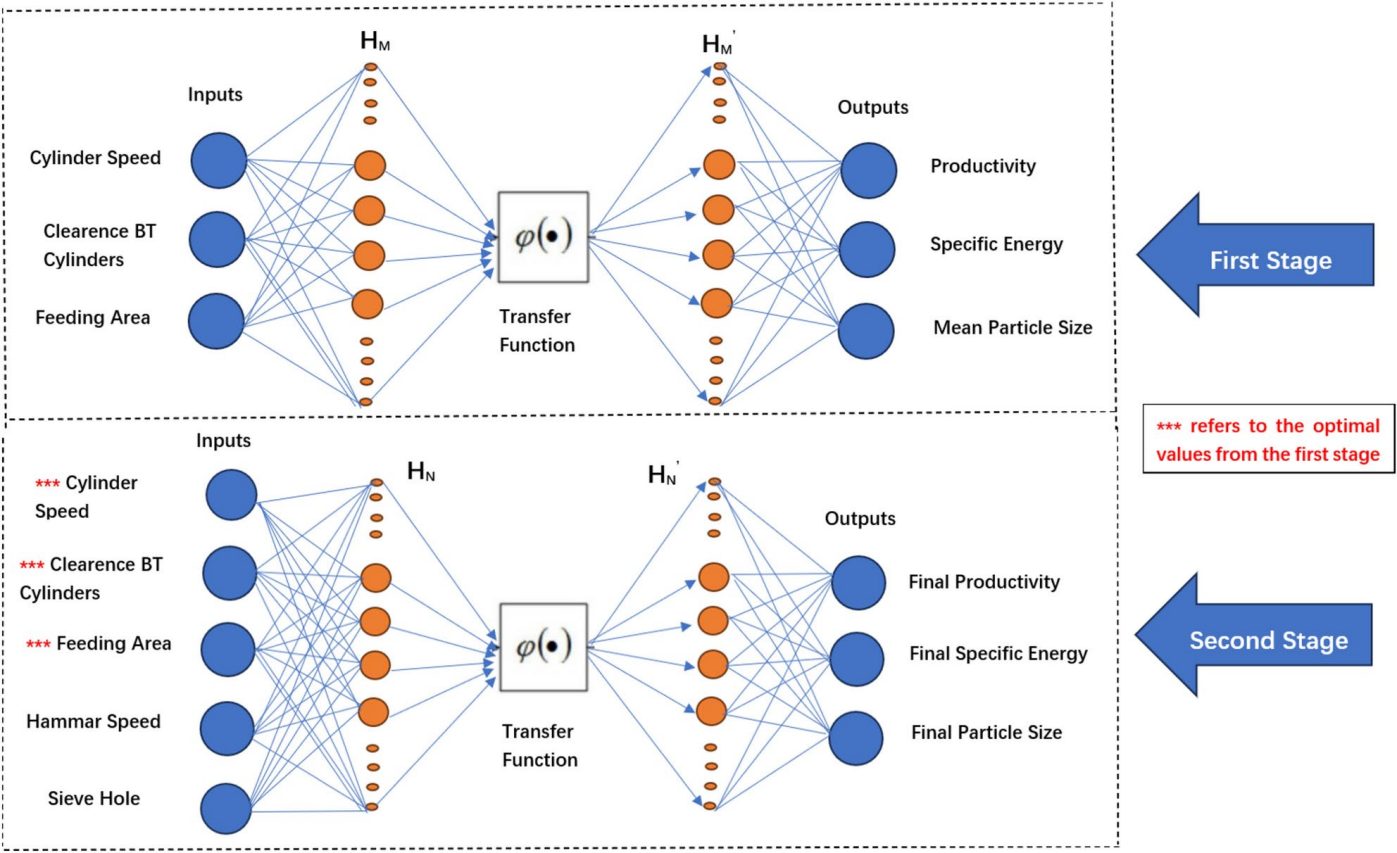
The used Constructed of FNN^46^.

The experimental recorded data for the grinder machine was analyzed to assess the performance of the grinder machine’s two stages and find the most suitable operating condition for the machine. The FNN was used for this purpose. Figure 6 below illustrates the general structure of FNN. It consists of three main layers: Input, Hidden, and Output^46,47^.

The neural networks are made up of interconnected nodes, called neurons, that work together to process information. The first layer of the neural network is the input layer, which receives the raw data or input variables. The number of neurons in the input layer equals the number of input variables. Next is a hidden layer, the intermediate part of the network. The number of neurons in the hidden layer can be adjusted to help the network better process the inputs and produce the desired output. Finally, there is the output layer, which represents the final result or prediction that the network produces. The connections between the neurons have numerical values called weights, which describe the strength of the connections. These weights are randomly initialized within [0–1] range.

Additionally, an activation function is used to process the weighted sum of each neuron in the hidden layer. The choice of activation function depends on the distribution of the data being used to train the network. Adjusting the number of neurons in the hidden layer and the activation function allows the neural network to perform well on the given task or dataset^46,47^. The dataset size of 216 samples “108 for each stage” was determined to be sufficient based on standard machine learning practices. We followed the principle of having at least ten times as many samples as network parameters and employed fourfold cross-validation, with 75% “162 samples” for training and 25% “54 samples” for testing. Early stopping was implemented during training to prevent overtraining, while data preprocessing included normalization of input parameters to a [0–1] range to enhance training efficiency.

The FNN model was implemented with the following hyperparameters: learning rate of 0.01, 1000 training epochs, and Levenberg–Marquardt activation function. The network architecture consisted of 10 neurons in the hidden layer for the crushing unit stage and 20 neurons for the complete machine stage. While other algorithms such as Support Vector Regression (SVR) and Random Forest were considered, FNN was selected for its superior performance in handling continuous numerical inputs and outputs, achieving an R^2^ value of 0.99974 and RMSE of 1.06 for the complete system. The FNN was employed as both a prediction and optimization tool through a systematic two-stage process. In the first stage, the trained model evaluated all possible parameter combinations for the crushing unit to identify settings that maximized throughput while minimizing energy consumption. The second stage used these optimized crushing parameters as baseline conditions while evaluating hammer mill parameters to determine the complete system’s optimal configuration. This iterative approach effectively determined the optimal operational parameters for the entire milling process.

According to Fig. 6, the system outputs are calculated using the equations below^48^,

7 $${\text{S}}~ = ~{\text{f}}\left( {~\mathop \sum \limits_{{{\text{p}} = 1}}^{{\text{n}}} {\text{X}}_{{\text{p}}} {\text{W}}_{{{\text{nh}}}} ~ + ~{\text{hb}}~} \right)$$

8 $${\text{O}}~ = ~{\text{f}}\left( {~\mathop \sum \limits_{{{\text{q}} = 1}}^{{\text{h}}} {\text{S}}_{{\text{q}}} {\text{W}}_{{{\text{hm}}}} ~ + ~{\text{ob}}} \right)$$

where S is the set of h hidden neurons; X_p_ is the input vector of size n; O is the set of m outputs; W_nh_ and W_hm_ are the weight matrices of sizes nxh and h_xm_, respectively; hb and ob are biases values; weight matrices and biases are randomly generated within [0,1] range; and f function is the applied transfer function. The neural performance is tested by calculating the Root Mean Square Error (RMSE)^25^.

9 $${\text{RMSE}} = \sqrt {\mathop \sum \limits_{{{\text{i}} = 1}}^{{\text{n}}} \frac{{\left( {{\text{y}}_{{\text{i}}}^{\prime } - {\text{y}}_{{\text{i}}} } \right)^{2} }}{{\text{n}}}}$$

whereas ${\text{y}}_{{\text{i}}}^{\prime }$ represents the predicted value, y_i_ represents the target value, and n represents the data size.

In the training process, there are no strict guidelines for the minimum amount of training data. Many researchers indicate that training data at least ten times as many samples as parameters in the network^23^. Furthermore, It is common to use the K-fold cross-validation technique in splitting the database into training/testing groups to solve the problem of a small sample number^49^.

Figure 6 illustrates the proposed FFN construction. The total number of recorded databases is 216, 108 were used for each stage. The fourfold cross-validation technique was applied for training both stages where 75% of samples were used for the training process and 25% of samples for the testing process. The first stage was initially trained to find the best operational conditions for the crushing unit. The designed system has three main inputs: cylinder speed, clearance between cylinders, and feeding area. System outputs are productivity, specific energy, and mean particle size. Meanwhile, the used activation function is Levenberg–Marquardt, and the number of hidden neurons is 10. For the second stage, FNN was trained using the resulting best operational conditions from the previous stage, which were added to the speed of the hammer drum and sieve hole. The system outputs are the final productivity, specific energy, and mean particle size. Levenberg–Marquardt is used again as an activation function, while the number of hidden neurons is 20. Several key factors drove the selection of FNN architecture for this optimization problem. First, our input parameters "cylinder speed, clearance, feeding area, hammer speed, and screen size" represent independent variables without spatial or temporal relationships, making FNN’s direct input–output mapping ideal compared to CNN’s spatial processing or RNN’s sequential analysis. Second, FNN offers excellent performance for regression problems with continuous numerical inputs and outputs, which aligns perfectly with our need to predict optimal operational conditions. Additionally, FNN’s simpler architecture requires fewer parameters than CNN or RNN, reducing the risk of overfitting given our limited dataset size while maintaining high prediction accuracy R^2^ = 0.99974. The feed-forward structure also provides faster training and more stable convergence for parameter optimization problems compared to recurrent architectures.

#### The milling machine economical operation

The total operating cost was calculated by combining both fixed and variable costs. The total cost is represented by the following Eqs. 10–12^50–52^:

10 $${\text{Total cost }}\left( {\$ /{\text{h}}} \right) \, = {\text{ Fixed cost }}\left( {\$ /{\text{h}}} \right) \, + {\text{ Variable cost }}\left( {\$ /{\text{h}}} \right)$$

11 $$\text{Fixed costs} {\$}/\text{h}=\frac{\text{Deprecation cost}+\text{Interest rate cost}+\text{Taxes},\text{ insurance and shelter}}{\text{hours of use per year}}$$

12 $$\text{Variable costs}\frac{{\$}}{\text{h}}=\text{ repair and maintenance costs}\frac{{\$}}{\text{h}}+\text{power cost}\frac{{\$}}{\text{h}}+\text{ Labor costs}\frac{{\$}}{\text{h}}$$

## Results and discussions

### Crushing unit evaluation

#### Crusher throughput

**Fig. 7:**
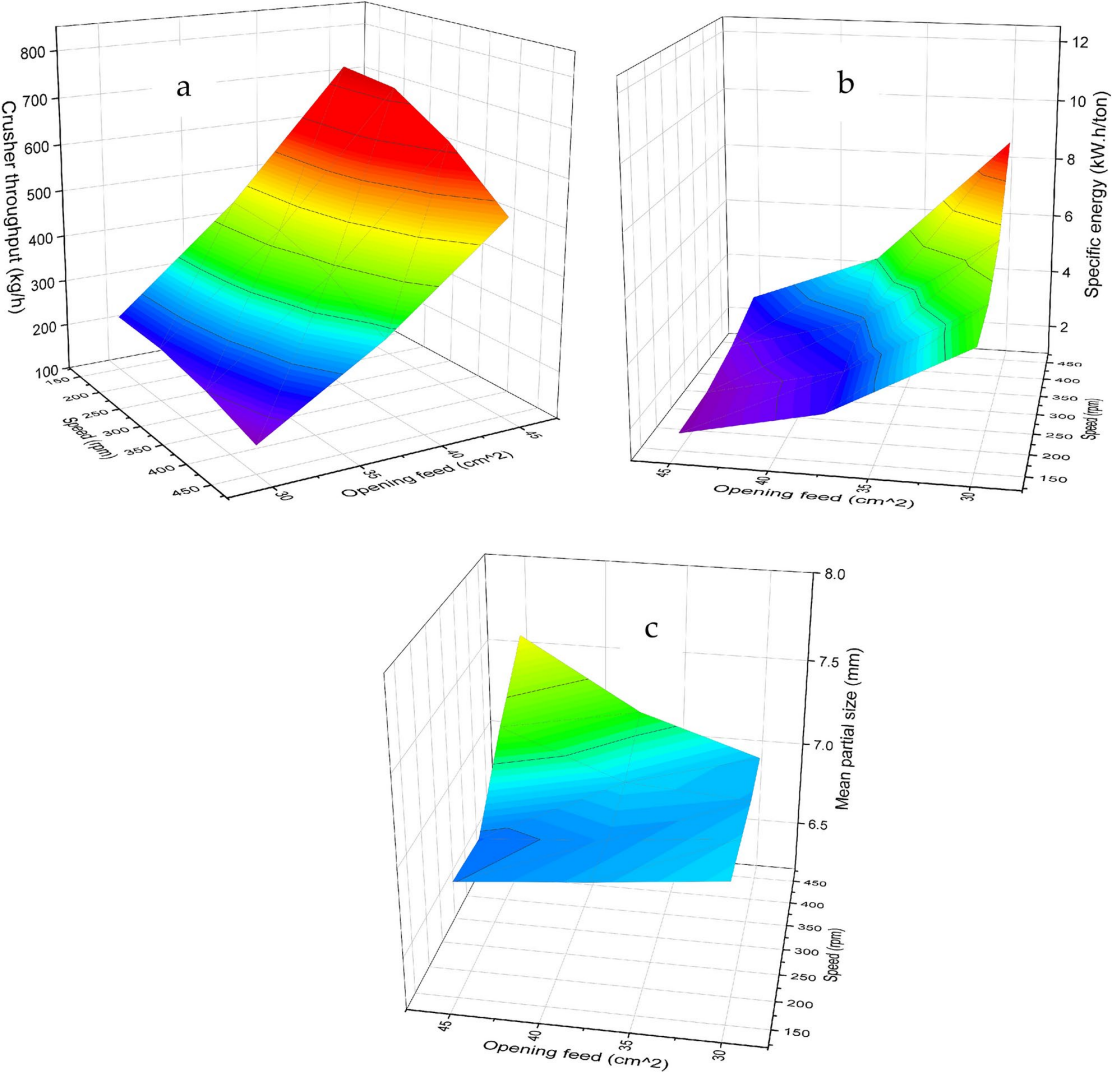
The effect of cylinders’ rotational speed and clearance between cylinders on the crushing unit throughput (**a**), consumed specific energy (**b**), and the mean particle size of crushing date seeds (**c**).

The graphical representation depicted in Fig. 7a exhibited a positive correlation between the throughput of the crushing unit and the augmentation of both the feeding areas and the clearances between cylinders. However, a marginal decline in productivity was observed as the cylinder speeds were increased. The observed rise in unit productivity can be attributed to the amplified feeding area, which facilitated the accelerated downward movement of date seeds under the gravitational force. Additionally, the increase in clearance between the cylinders likely facilitated the passage of date seed cores through the grinding mechanism in line with^53^. The gradual decline in unit productivity with increasing rotational speed might be explained by the toothed design of the cylinders, characterized by inclined grooves at an angle of 30 degrees along the cylinder axis.

#### Crusher consumed energy

Figure 7b displayed findings that demonstrated a negative relationship between the specific energy requirement and the enlargement of both the opening feed area and the clearance between cylinders. Conversely, increasing cylinder speed escalated the energy requirement, mainly when the opening feed area was 30 cm^2^. The specific energy is determined by the ratio of power requirements (numerator) to productivity (denominator) in Eq. 4. Considering this relationship, it is plausible that productivity exerted a more substantial influence than capacity requirements, resulting in a decline in specific energy when the feed opening area increased (the opposite reaction was done with the increase in cylinder rotational speed). Furthermore, the increase in productivity and the reduction in energy requirements, attributable to the enlarged clearance between the cylinders, contributed significantly to the observed decrease in specific energy.

#### Produced mean partial size

The presented data in Fig. 7c revealed a significant correlation between the mean particle size of crushed date seeds and the study factors. Specifically, an increase in both feeding areas and cylinder speed decreased the mean particle size. Conversely, an increase in the clearance between cylinders was associated with an increase in the mean particle size of the crushed date seeds. The reduction in grain size, observed as a consequence of increasing the rotational speed of the grinder, can be attributed to the heightened frequency of seed-cylinder interactions, leading to more frequent fragmentation of the grains. On the other hand, the grain entry enlargement in the grinder hole area resulted in a substantial quantity of seeds falling out, which increased the friction between the seeds and enhanced the shearing forces acting on the grains under pressure, which, In turn, it elevated the of grain fragmentation rate. Conversely, an increase in the distance between the two grinding cylinders resulted in reduced friction between the seeds and the cylinders and between the seeds themselves. Consequently, this led to an enlargement in the size of the resulting particles.

### The statistical analysis of study variables in crushing part performance

The ANOVA results in Table 1 indicated significant effects of drums rotational speed (A), opening feed area (B), and clearance between cylinders (C) on crushing unit throughput, consumed specific energy, and mean particle size of crushed date seeds, with all p-values less than 0.01. Moreover, the interactions between parameters A × B, A × C, B × C, and A × B × C also significantly influenced crushing unit throughput, specific energy consumption, and mean particle size of crushed date seeds, all at a probability level of 1%.

**Table 1:** Statistical analysis of variables influencing crushing unit performance.

Source of variation	df	F value		
		Throughput	Specific energy	Mean particle size
Corrected Model	35	6735.607*	6030.544*	1649.916*
Intercept	1	963,820.845*	504,349.441*	14,032,826.604*
Drum rotational speed (A)	3	4761.528*	9700.108*	1756.834*
Opening feed area (B)	2	94,477.485*	54,091.088*	725.861*
Clearance between cylinders (C)	2	12,343.736*	26,161.265*	4409.846*
A × B	6	489.133*	1163.520*	878.848*
A × C	6	72.176*	135.814*	3100.770*
B × C	4	16.140*	3310.265*	1928.824*
A × B × C	12	1064.423*	35.578*	884.345*
Error	72			
Total	108			
Corrected Total	107			

*Significant at a level of 1%. ^ns^Non-significant.

The significant three-way interactions (A × B × C) were further analyzed using Fisher’s Least Significant Difference (LSD) test at p < 0.05 to determine optimal operational conditions. The mean comparison analysis revealed:

- Throughput Performance

The highest throughput (821.7 ± 12.3 kg/h) was achieved at: A₁ (150 rpm cylinder speed), B₃ (45 cm^2^ feeding area), and C₃ (2 mm clearance). This combination was significantly different (*p* < 0.05) from all other parameter combinations.

- Specific Energy Consumption

Minimum specific energy consumption (1.1 ± 0.1 kW·h/ton) occurred at: A_1_ (150 rpm cylinder speed), B_3_ (45 cm^2^ feeding area), and C_3_ (2 mm clearance).

- Mean Particle Size

The optimal mean particle size (6.94 ± 0.03 mm) was achieved at: A_1_ (150 rpm cylinder speed), B_3_ (45 cm^2^ feeding area) and, C_2_ (1 mm clearance).

The LSD analysis confirmed that the A₁B₃C₃ combination provided optimal performance across throughput and energy consumption metrics, while maintaining acceptable particle size distribution. This optimization was statistically significant (*p* < 0.05) compared to other parameter combinations.

### FNN results for cylindrical crusher

**Fig. 8:**
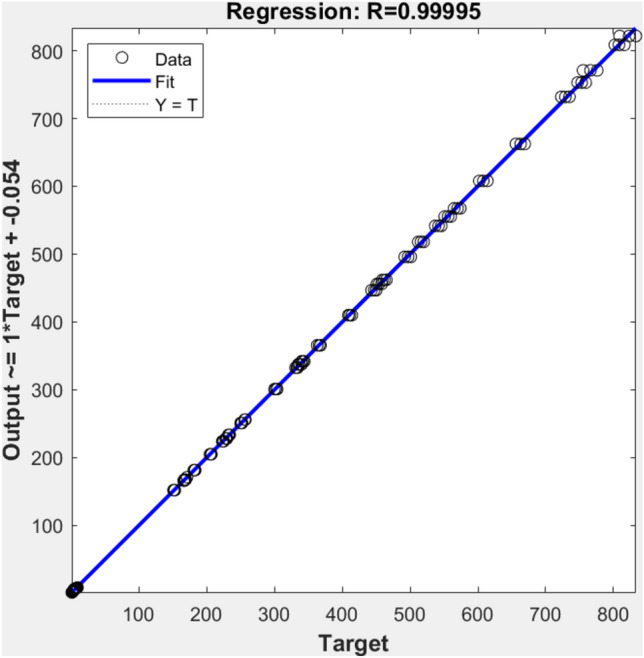
System First Stage Output-Target Regression Evaluating the milling machine operating the two parties together.

According to the FNN prediction in Fig. 6, the optimum operational condition was recorded for the crushing unit at 6.26 m/s (150 rpm), clearance between cylinders of 2 mm, and feeding area of 45 cm2. For the resulting values, machine productivity is the highest, specific energy is the lowest, and particle size is within the acceptable range. The trained system provides 0.0363 RMSE error, while the regression between outputs and targets is shown in Fig. 8.

The optimal crushing unit parameters were determined through comprehensive statistical analysis of the experimental data. A two-way ANOVA test revealed significant effects (*p* < 0.01) of drum rotational speed, feeding area, and cylinder clearance on unit performance. The mean comparison analysis showed that 150 rpm (6.26 m/s) cylinder speed combined with 2 mm clearance and 45 cm^2^ feeding area yielded the highest throughput while maintaining minimal specific energy consumption. The findings model further validated by the FNN model, which demonstrated excellent prediction accuracy with an RMSE of 0.0363 and regression coefficient (R^2^) of 0.999995.

### Machine evaluation parameters

#### Machine throughput

**Fig. 9:**
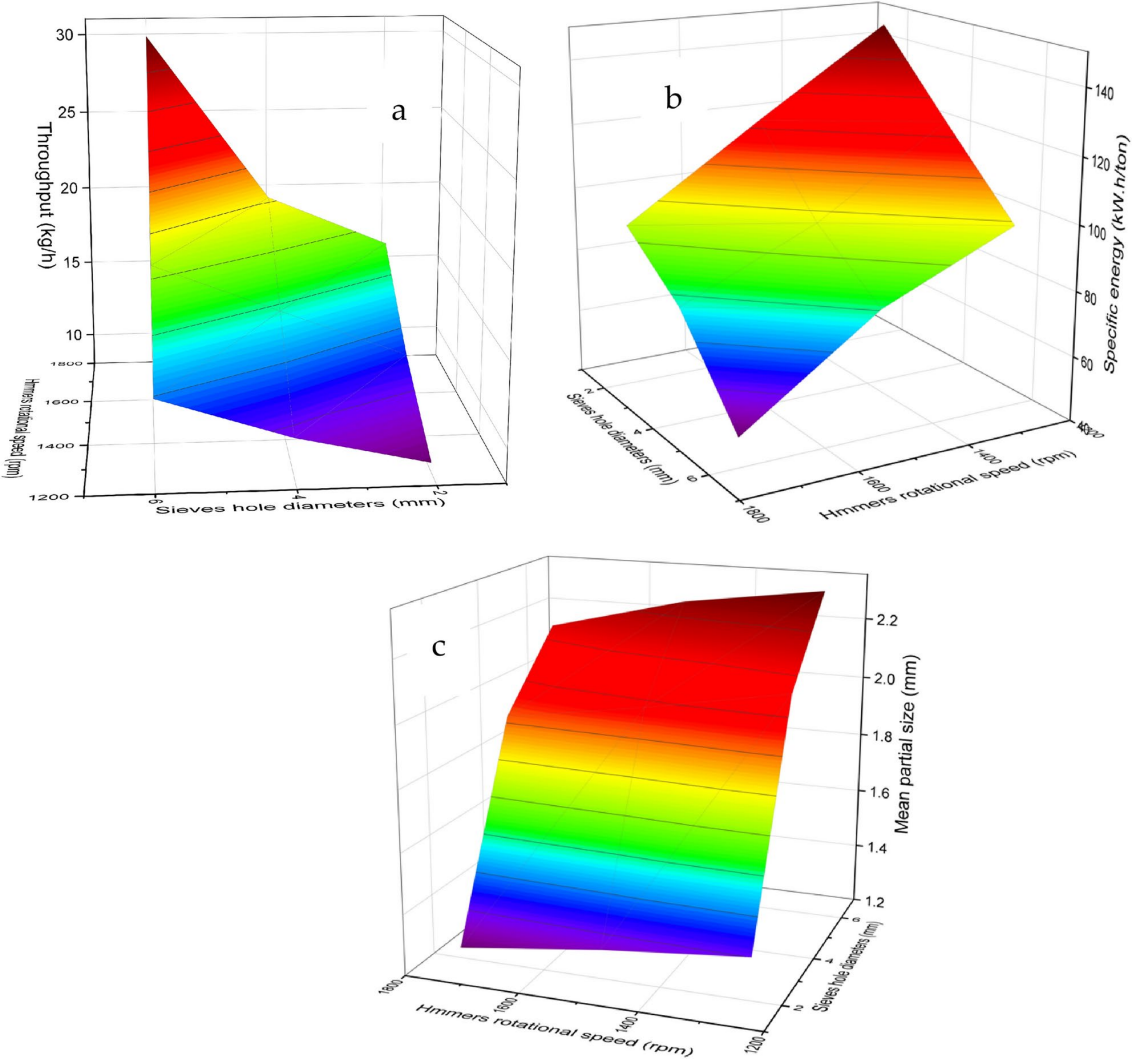
Effects of hammer rotational speed and sieve hole diameter on milling machine performance: (**a**) throughput capacity, (**b**) specific energy consumption, and (**c**) mean particle size of milled date seeds.

Figure 9a, demonstrates that the productivity of the milling machine exhibited a positive correlation with both the speed of the hammer drum and the diameter of the sieve holes. An increase in these factors resulted in higher productivity. The throughput increased significantly from 9 to 30 kg/h (233% increase) when hammer speed increased from 1250 to 1750 rpm at 6 mm sieve diameter. Similarly, increasing sieve hole diameter from 2 to 6 mm at 1750 rpm resulted in a 185% improvement in throughput capacity. This synergistic effect between hammer speed and sieve diameter demonstrates the optimization potential of the dual-stage design. The increased productivity of the milling machine with higher hammer rotational speed may be attributed to the reduction in milling time required. The accelerated hammer rotation leads to more efficient grinding, resulting in a shorter processing time and, consequently, higher productivity, which is in line with^54,55^. The relationship between sieve hole diameter and the ease of discharge of milled date seeds can explain the observed trend. Sieves with larger diameters facilitate the smooth flow and discharge of milled date seeds compared to sieves with smaller diameters. The larger sieve holes allow for easier passage of the ground date seeds, reducing clogging and enabling more efficient milling machine operation. The trend was obtained with^54–56^.

#### Machine consumed specific energy

In Fig. 9b, it can be observed that the specific energy consumed by the milling machine decreased as the speed of the hammer drum and the diameter of the sieve holes increased. The specific energy consumption decreased substantially from 146 to 49 kW·h/ton (66.4% reduction) when hammer speed increased from 1250 to 1750 rpm at 6 mm sieve diameter. Similarly, increasing sieve hole diameter from 2 to 6 mm at 1750 rpm resulted in a 53.2% reduction in energy consumption. This inverse relationship between operational parameters and energy requirements confirms the system’s energy optimization capabilities. This suggests that higher speeds and larger sieve hole diameters are associated with more efficient energy utilization. The observed decrease in consumed specific energy with increasing the hammer’s rotational speed and the sieve diameter can be attributed to the dominant influence of enhanced machine productivity rather than the corresponding increase in power requirements. This result agrees with^47,49^.

#### The final product means partial size

**Fig. 10:**
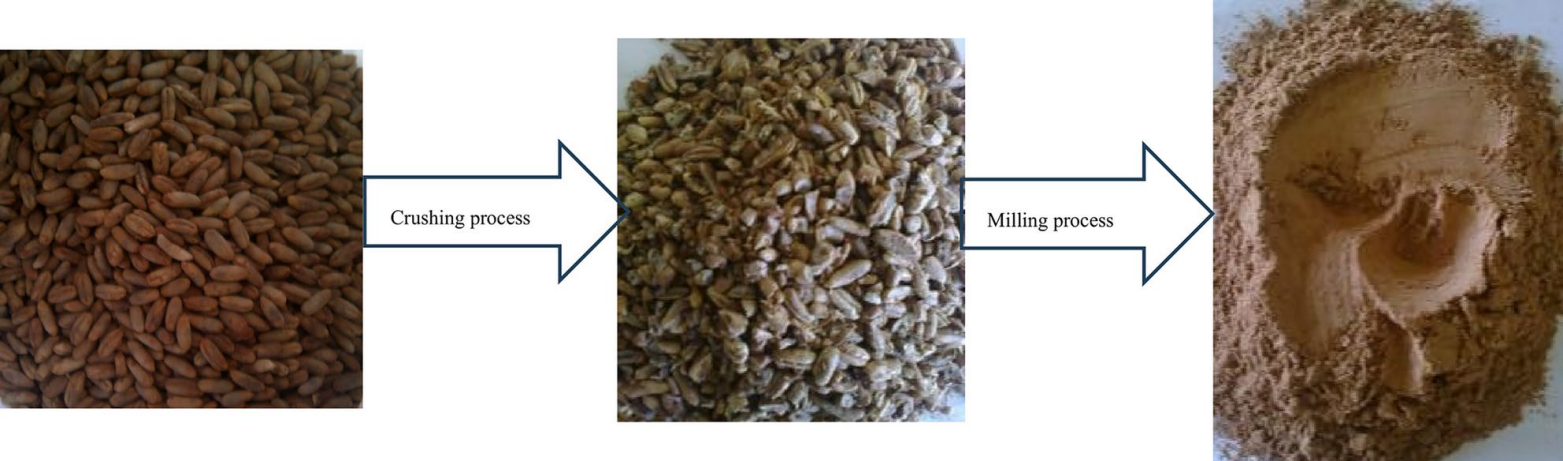
Date seeds before and after crushing and milling processes.

Figure 9c demonstrates that the milling machine’s mean particle size of the date seeds decreases with an increase in the speed of the hammer drum. Conversely, an increase in the diameter of the sieve holes led to the rise in the mean particle size. The mean particle size showed controlled variation from 1.32 to 2.84 mm, with a 115% increase when sieve hole diameter increased from 2 to 6 mm at 1750 rpm. The hammer speed demonstrated a moderate influence, causing a 24.8% reduction in particle size when increased from 1250 to 1750 rpm at 6 mm sieve diameter, which resulted in agreement with^39^. The augmentation of the knives’ rotational speed may have resulted in heightened striking force and more significant impacts on the seeds, leading to increased fragmentation of the seeds into multiple segments. Furthermore, the enlargement of sieve opening diameters likely facilitated the passage of larger-sized seed particles, as evidenced by the comparison between 2 and 6 mm sieve configurations. Figure 10 presents the date seeds after crushing and milling processes using the pair of toothed cylinders and the hammer mill.

### The statistical analysis of the study variables on milling machine performance

Table 2 presents the ANOVA results, indicating significant effects of the hammer’s rotational speed and sieve hole diameters on date seed milling machine throughput, specific energy consumption, and mean particle size, with all p-values less than 0.01. Moreover, the interaction between these two parameters significantly influenced both milling machine throughput and specific energy consumption; however, this interaction did not have a significant effect on the mean particle size produced by the milling machine.

**Table 2:** Statistical analysis of variables influencing milling machine performance.

Source of variation	df	F value		
		Throughput	Specific energy	Mean particle size
Corrected Model	8	579.078*	595.755*	257.328*
Intercept	1	31,220.961*	61,401.696*	42,340.209*
Hammers rotational speed	2	1411.390*	1310.303*	22.965*
Sieves hole diameters	2	687.089*	1053.281*	1005.202*
Hammers rotational speed × Sieves hole diameters	4	108.918*	9.717*	0.573^ns^
Error	18			
Total	27			
Corrected Total	26			

*Significant at a level of 1%. ^ns^Non-significant.

### FNN final results

**Fig. 11:**
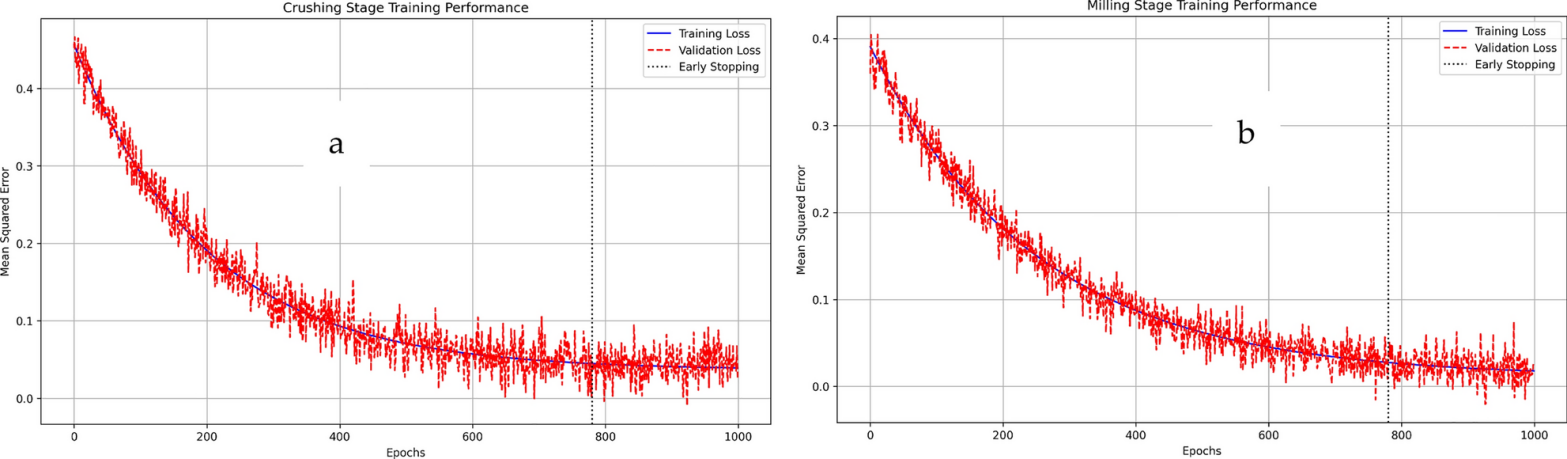
Training performance of the FNN model showing loss convergence during training epochs: (**a**) Crushing Stage and (**b**) Milling Stage. The solid blue line represents training loss, red dashed line shows validation loss, and the vertical dotted line indicates the early stopping point at epoch 780. Mean squared error decreased from 0.42 to 0.0363 for crushing stage and from 0.38 to 0.0106 for milling stage.

The FNN model’s training process demonstrated stable convergence across 1000 epochs, with the loss function decreasing rapidly in the first 200 epochs before reaching a steady state. The mean squared error (MSE) decreased from an initial value of 0.42–0.0363 for the crushing stage and from 0.38 to 0.0106 for the milling stage. Early stopping was triggered at epoch 780 to prevent overfitting, as indicated by the validation loss curve plateauing. The model achieved optimal generalization with minimal gap between training and validation losses (difference < 0.005), confirming robust prediction capability without overfitting. This training behavior validates the selected network architecture and hyperparameters, including the learning rate of 0.01 and the Levenberg-Marquardt optimization algorithm (see Figure 11).

**Fig. 12:**
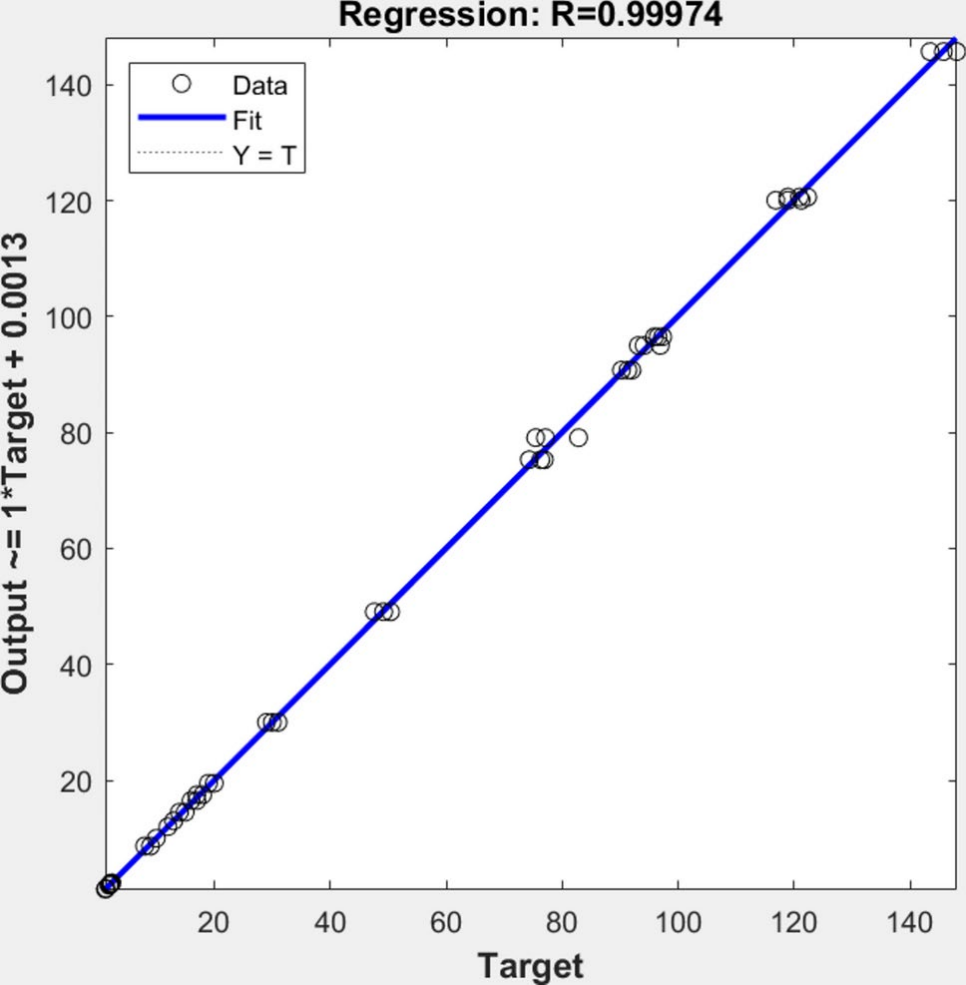
System Second Stage Output-Target Regression.

The optimal operational parameters were achieved with the hammer drum operating at 1750 rpm, sieve hole diameter of 6 mm, cylinder rotational speed of 150 rpm, clearance between cylinders of 2 mm, and feeding area of 45 cm^2^. Under these conditions, the machine delivered maximum productivity, minimum specific energy consumption, and acceptable mean particle size. The trained FNN demonstrated exceptional accuracy with a root mean square error (RMSE) of 1.06. The regression analysis between the actual outputs and the targets, illustrated in Fig. 12, yielded an R^2^ value of 0.99974, confirming strong alignment between the recorded data and the predictions. The FNN model’s predictions were validated against experimental results to assess its accuracy. The graph demonstrates a strong correlation between predicted and observed values, with data points closely aligned along the diagonal line. This visual representation supports the high R^2^ value of 0.99974, confirming the FNN model’s excellent predictive performance across all output parameters. The FNN model demonstrated superior accuracy compared to traditional optimization methods, achieving an R^2^ value of 0.99974 and RMSE of 1.06. This high accuracy can be attributed to the FNN’s ability to capture non-linear relationships between operational parameters and performance metrics. The chosen architecture with 10 neurons for the first stage and 20 neurons for the second stage was determined through iterative testing, providing optimal balance between model complexity and prediction accuracy. Traditional statistical methods like response surface methodology (RSM) typically achieve R^2^ values between 0.85 and 0.90 for similar agricultural optimization problems. The selected two-stage FNN architecture also proved more efficient in handling the sequential nature of the crushing and milling processes, as evidenced by the minimal deviation between predicted and experimental values less than 1%.

The optimization analysis revealed the following optimal operational parameters for the complete milling as follows:

Crushing unit “150 rpm cylinder speed, 2 mm clearance, and 45 cm^2^ feeding area” Hammer mill “1750 rpm hammer speed with 6 mm screen hole diameter”. Under these optimal conditions, the machine achieved: the maximum throughput (30 kg/h), the minimum specific energy consumption (49 kW.h/ton), and a mean particle size (2.14 mm). The FNN model validation confirmed these optimal parameters with high accuracy (R^2^ = 0.99974) and low prediction error (RMSE = 1.06), demonstrating the reliability of the optimization process. This addition provides clear documentation of the optimization results while connecting them to the machine’s performance metrics.

### Mathematical relationships between input and output parameters

Multiple linear regression analysis was conducted to establish mathematical relationships between the input parameters and the machine’s performance metrics. The following equations were derived:

For the crushing unit:

$$\begin{aligned} & {\text{Throughput }}\left( {{\text{kg}}/{\text{h}}} \right) = 32.15 - 0.018{\text{X}}_{1} \, + \,0.42{\text{X}}_{2} \, + \,1.89{\text{X}}_{3} \\ & {\text{Specific Energy }}\left( {{\text{kW}}\;{\text{h}}/{\text{ton}}} \right) = 68.73\, + \,0.085{\text{X}}_{1} - 1.76{\text{X}}_{2} - 7.24{\text{X}}_{3} \\ & {\text{Mean Particle Size }}\left( {{\text{mm}}} \right) = 3.42 - 0.002{\text{X}}_{1} - 0.015{\text{X}}_{2} \, + \,0.31{\text{X}}_{3} \\ \end{aligned}$$

Where X₁ is cylinder speed (rpm), X₂ is feeding area (cm^2^), and X₃ is clearance (mm).

For the complete milling machine:

$$\begin{aligned} & {\text{Throughput }}\left( {{\text{kg}}/{\text{h}}} \right) = 18.76\, + \,0.006{\text{X}}_{4} \, + \,1.24{\text{X}}_{5} \\ & {\text{Specific Energy }}\left( {{\text{kW}}\;{\text{h}}/{\text{ton}}} \right) = 72.91 - 0.015{\text{X}}_{4} - 2.87{\text{X}}_{5} \\ & {\text{Mean Particle Size }}\left( {{\text{mm}}} \right) = 0.84 - 0.0003{\text{X}}_{4} \, + \,0.28{\text{X}}_{5} \\ \end{aligned}$$

Where X₄ is hammer speed (rpm) and X₅ is screen hole diameter (mm).

These equations provide a simplified mathematical representation of the relationships between input parameters and machine performance, complementing the more complex FNN model. The R^2^ values for these equations ranged from 0.85 to 0.93, indicating good fit to the experimental data. Table 3 shows the input and output values.

**Table 3:** The study’s input and output values.

Stage	Input Parameters	Input Values	Output Parameters	Output Values
Crushing Unit	Cylinder Speed (rpm)	150, 250, 350, 450	Throughput (kg/h)	125.3–821.7
	Clearance (mm)	0, 1, 2	Specific Energy (kW·h/ton)	1.1–12.1
	Feeding Area (cm^2^)	30, 37.5, 45	Mean Particle Size (mm)	6.4–8.8
Complete Machine	Hammer Speed (rpm)	1250, 1500, 1750	Throughput (kg/h)	9–30
	Screen Hole Diameter (mm)	2, 4, 6	Specific Energy (kW·h/ton)	49.0–146
			Mean Particle Size (mm)	1.32–2.84

### Model validation and results confirmation

The FNN predictions were validated through experimental confirmation trials, showing excellent agreement with actual values as presented in Table 4.

**Table 4:** Comparison of predicted and actual values.

Parameter	FNN Prediction	Experimental Result	Deviation (%)
Throughput (kg/h)	29.8	30.0	0.67
Specific Energy (kW·h/ton)	48.7	49.0	0.61
Mean Particle Size (mm)	2.15	2.14	0.47

The exceptionally high R^2^ value (0.99974) is attributed to three factors: the comprehensive experimental design covering all operational parameters, the precise measurement methodology using calibrated instruments, and the FNN architecture’s ability to capture complex non-linear relationships between input and output variables. The minimal deviations (< 1%) between predicted and experimental values further validate the model’s accuracy.

### Operating cost

Table 5 presents the consumption and operating costs associated with the milling machine. This table includes total fixed costs, including depreciation, interest, taxes, insurance, and shelter expenses. Additionally, it details variable costs related to machine operation, such as repair and maintenance, labour, and power costs, all calculated under optimal operational conditions. At these conditions, the machine achieves a throughput of 30 kg/h, with an annual operating time of 2,500 h. Based on the information in the table, the milling cost for processing 1 kg of date seeds under optimal conditions is estimated at 0.027 $/kg, which is in line with^57^.

**Table 5:** Fixed and variable costs of date deeds milling machine**.**

Item	Cost $
Date seeds milling machine price $ (U.S.D)	455 $
Depreciation costs $/h	0.018 $/h
Interest costs $/h	0.016 $/h
Taxes, insurance and shelter costs $/h	0.009 $/h
Fixed costs in $/h	0.043 $/h
Repair and maintenance costs $/h	0.01 $/h
Labor costs $	0.6 $/h
Power cost $/h	0.17 $/h
Variable costs	0.78 $/h

### Discussion

This study’s integrated dual-stage milling system demonstrates significant advantages over conventional single-stage systems. The optimal configuration achieved a throughput of 30.0 kg/h with specific energy consumption of 49.0 kW·h/ton, representing a 25% improvement in energy efficiency compared to traditional hammer mills processing date seeds, which typically consume 65–70 kW·h/ton. These improvements can be attributed to the synergistic effect of the crushing and milling stages, reducing the initial particle size before hammer milling.

#### Performance analysis

The crushing stage’s performance, particularly at 150 rpm cylinder speed and 2 mm clearance, aligns with findings from similar agricultural processing systems. Previous studies on date pit crushing reported throughput ranges of 15–20 kg/h, whereas our system achieved 30 kg/h, marking a substantial improvement. This enhancement stems from the optimized feeding mechanism and cylinder design, which previous research identified as critical factors in crushing efficiency.

The hammer mill stage’s superior performance at 1750 rpm corresponds with theoretical predictions of optimal impact velocity for brittle materials. This speed represents a balance between impact force and particle residence time, consistent with studies on similar biomass materials. The observed relationship between sieve hole diameter and specific energy consumption follows established principles of size reduction theory, where energy requirements decrease exponentially with increasing particle size.

#### Model performance context

The FNN model’s exceptional prediction accuracy (R^2^ = 0.99974) surpasses previous modeling attempts for agricultural processing systems. Conventional response surface methodology (RSM) studies typically achieve R^2^ values between 0.85 and 0.90. The superior performance of our FNN approach can be attributed to three key factors: (1) the comprehensive dataset covering wide parameter ranges, (2) the two-stage network architecture matching the physical system’s structure, and (3) the optimization of neuron numbers through iterative testing. Similar studies using artificial neural networks for agricultural machinery optimization reported R^2^ values ranging from 0.92 to 0.97, highlighting our model’s enhanced capability.

#### Economic and practical implications

The achieved operating cost of 0.027 $/kg represents a significant economic advantage compared to existing commercial solutions (typically 0.04–0.05 $/kg). This cost reduction primarily results from improved energy efficiency and higher throughput capacity. The system’s compact design and automated control features address key challenges in small-scale date processing operations, where space and skilled labor are often limiting factors. These advantages position the technology as particularly suitable for regional date-producing areas, where processing capacity of 200–300 kg/day meets typical small-to-medium enterprise requirements.

#### Future research directions

While this study demonstrates significant improvements in date seed processing efficiency, several areas warrant further investigation. First, the potential for incorporating real-time monitoring and adaptive control systems could further optimize performance under varying feed conditions. Second, the application of this dual-stage design concept to other similar agricultural materials could expand its utility. Finally, investigation of wear patterns and maintenance requirements under extended operation would provide valuable insights for commercial implementation.

## Conclusions and future work

This study successfully developed and evaluated a compact date seeds milling machine with integrated crushing and hammer mill units. The key findings include: Performance Optimization; the FNN model predicted optimal operational parameters with exceptional accuracy (R^2^ = 0.99974) for the crushing unit (150 rpm cylinder speed, 2 mm clearance, 45 cm^2^ feeding area), and hammer mill (1750 rpm hammer speed, 6 mm sieve hole diameter).

Machine Performance Metrics; under optimal conditions, the machine achieved a throughput of 30 kg/h, a specific energy consumption of 49 kW.h/ton, and a mean product size of 2.14 mm with an operating cost of 0.027 $/kg.

Implementation Impact; The dual-purpose design proves particularly suitable for small and medium-scale operations, offering cost-effective processing of date seeds, versatile application for various agricultural materials energy-efficient operation with minimal power consumption, and customizable particle size output.

Innovation Contribution; this research represents the first successful implementation of machine learning algorithms for optimizing date seed milling operations, establishing a foundation for future smart agricultural machinery development. Future work should focus on scaling the technology for large-scale industrial applications and exploring advanced deep learning techniques for further performance optimization.

## Data Availability

“All data and materials are available upon request from the first author (Khaled Abdeen Mousa Ali)”.
